# Healthcare-seeking behaviour in case of influenza-like illness in the French general population and factors associated with a GP consultation: an observational prospective study

**DOI:** 10.3399/bjgpopen17X101253

**Published:** 2017-12-13

**Authors:** Matthieu Ariza, Caroline Guerrisi, Cécile Souty, Louise Rossignol, Clément Turbelin, Thomas Hanslik, Vittoria Colizza, Thierry Blanchon

**Affiliations:** 1 Graduate Researcher, Sorbonne Universités, UPMC Univ Paris 06, INSERM, Institut Pierre Louis d'Epidémiologie et de Santé Publique, Paris, France; 2 Epidemiologist, Sorbonne Universités, UPMC Univ Paris 06, INSERM, Institut Pierre Louis d'Epidémiologie et de Santé Publique, Paris, France; 3 Biostatistician, Sorbonne Universités, UPMC Univ Paris 06, INSERM, Institut Pierre Louis d'Epidémiologie et de Santé Publique, Paris, France; 4 GP, Sorbonne Universités, UPMC Univ Paris 06, INSERM, Institut Pierre Louis d'Epidémiologie et de Santé Publique, Paris, France; 5 GP, Sorbonne Universités, UPMC Univ Paris 06, INSERM, Institut Pierre Louis d'Epidémiologie et de Santé Publique, Paris, France; 6 Internist Physician, UFR des Sciences de la Santé Simone-Veil, Université de Versailles Saint-Quentin-en-Yvelines, Versailles, France; 7 Internist Physician, Service de Médecine Interne, Hôpital Ambroise Paré, Assistance Publique — Hôpitaux de Paris, APHP, Boulogne-Billancourt, France; 8 Internist Physician, Sorbonne Universités, UPMC Univ Paris 06, INSERM, Institut Pierre Louis d'Epidémiologie et de Santé Publique, Paris, France; 9 Senior Researcher, Institute for Scientific Interchange, ISI Foundation, Turin, Italy; 10 Senior Researcher, Sorbonne Universités, UPMC Univ Paris 06, INSERM, Institut Pierre Louis d'Epidémiologie et de Santé Publique, Paris, France; 11 Public Health Physician, Sorbonne Universités, UPMC Univ Paris 06, INSERM, Institut Pierre Louis d'Epidémiologie et de Santé Publique, Paris, France

**Keywords:** influenza-like illness, healthcare seeking behaviour, general population, web-based study, general practice

## Abstract

**Background:**

GP consultation rates for influenza-like illness (ILI) are poorly known in France and there is a paucity of literature on this topic. In the few articles that have been published, the results are heterogeneous.

**Aim:**

The aim of the present study was to estimate the proportion of ILI inducing a GP consultation, and to assess its determinants.

**Design & setting:**

Participants of a French web-based cohort study who reported ≥1 ILI episode between 2012 and 2015 were included. Sociodemographic characteristics, access to health care, and health status variables were collected.

**Method:**

Healthcare-seeking behaviour was analysed and factors associated with a GP consultation identified using a conditional logistic regression.

**Results:**

Of the 6023 ILI episodes reported, 1961 (32.6%) led to a GP consultation, with no difference between those at risk of influenza complications and those not (*P* = 0.42). A GP consultation was more frequent for individuals living in a rural area (odds ratio [OR] = 1.21, 95% confidence interval [CI] = 1.02 to 1.43); those with a lower educational level (OR = 1.43, 95% CI = 1.18 to 1.74); those using the internet to find information about influenza (OR = 1.63, 95% CI = 1.30 to 2.03); patients presenting with worrying symptoms (fever, cough, dyspnoea, sputum, or asthenia); patients having a negative perception of their own health status (OR = 1.51, 95% CI = 1.07 to 2.13; and those having declared a personal doctor (OR = 2.86, 95% CI = 1.72 to 4.76). A GP consultation was less frequent for individuals using alternative medicine (OR = 0.68, 95% CI = 0.58 to 0.78).

**Conclusion:**

This study allows the identification of specific factors associated with GP consultation for an ILI episode. These findings may help to coordinate health information campaigns and to raise awareness, especially among individuals at risk of influenza complications.

## How this fits in

Characterising the healthcare-seeking behaviour of the general population in cases of ILI is important to gain a better knowledge of influenza's disease burden, and to identify factors associated with GP consultation. As expected, only a minority of ILI episodes were followed by a consultation with a GP (32.6%). Individuals at risk of serious influenza complications did not consult more than others and did not receive antiviral treatment more often. These findings suggest improved health information campaigns could raise awareness among both individuals at risk and GPs.

## Introduction

Each year in France, between 700 000 and 4.8 million individuals consult their GP for ILI, representing 1–8% of the French population.^1–2^ Although recovery is generally rapid, influenza can cause serious complications, especially in those aged >65 years old, pregnant women, individuals with chronic illnesses, or obesity.^3,4^ Mortality due to influenza during seasonal epidemics is estimated in France to be 1620–11 400 deaths per year,^5^ and in the world 250 000–500 000 deaths per year.^6^

Influenza epidemiological surveillance is traditionally carried out by health professionals, organised in national networks (such as the French Sentinelles network^7^ or the Italian Influenza Surveillance network^8^), and brought together under the coordination of the World Health Organization (WHO) in the Global Influenza Surveillance and Response System (GISRS).^9^ However some patients with ILI do not seek medical care and are not taken into account in these national networks, leading to an underestimation of the ILI rate among the general population. Cohort studies are thus needed to estimate this proportion of patients who do not seek medical care in case of ILI.^10 ^Several different results have been obtained reflecting the proportion of patients who do seek medical care in case of ILI, ranging from 4% in an Italian internet-based study up to 85% in an Israeli phone survey.^10–16 ^ Such disparities can be explained by the heterogeneity of social security systems among the analysed countries, different study designs, and different ILI definitions. In France, only two studies have been conducted on this topic. The first one was conducted on households which were recruited when one member made a visit to a GP for ILI, and the second study was performed during the A(H1N1)pdm09 influenza pandemic. The proportions of GP consultations for ILI were estimated to be 57% and 62%, respectively.^17, 18^ However, these findings cannot be extrapolated to the general population during a seasonal influenza epidemic because of the specific conditions under which they were performed: the first with a focus on a specific subgroup, and the second during an influenza pandemic. Moreover, evaluation of healthcare-seeking behaviour in specific groups of individuals, such as those at risk of influenza complications, was not performed. It is important to know if these individuals consult a GP and benefit effectively from an antiviral treatment in case of recommendation. Neuraminidase inhibitor treatment is recommended as soon as possible by French public health authorities,^19^ as in most other countries,^20,21^ for patients with ILI who: a) are at risk of influenza complications (defined as patients with ≥1 of the following characteristics: age ≥65 years, underlying chronic disease, obesity [defined as BMI ≥40 kg/m^2^], or pregnancy); b) are hospitalised; or c) have severe, complicated, or progressive illness. Currently only neuraminidase inhibitors (oseltamivir and zanamivir) may be prescribed; amandatanes are no longer recommended because of viral resistance.^22^

Characterising healthcare-seeking behaviour even further is important in order to identify the barriers to GP consultations in case of ILI, and to improve information campaigns and healthcare accessibility. A random-digit-dialling phone survey in the US has shown that GP consultation is associated with female sex, age >65 years, chronic illness, having a health insurance, and reporting a personal doctor.^13,14^ No factor was significantly associated with GP consultation in an Israeli study.^12^ Overall, outside the ILI context, a review of the international literature has shown that the choice to consult a physician is influenced by various factors linked to economical, sociodemographic, health condition, behavioural, or geographic characteristics.^23^

The objectives of this study were to characterise healthcare-seeking behaviour for ILI among the general population in France, and to analyse the associated factors.

## Method

This observational prospective study is based on data from the web-based GrippeNet.fr cohort study (www.grippenet.fr). GrippeNet.fr was implemented in France in January 2012 by the French National Institute for Health and Medical Research (Inserm), Pierre and Marie Curie University, and Public Health France. One of the purposes is to collect data on ILI directly from the population during influenza season (from November to April). GrippeNet.fr is part of the European consortium Influenzanet (https://www.influenzanet.eu/).^24^

The inclusion criteria to participate to GrippenNet.fr are living in France and having an email address. Participation is voluntary and anonymous after an online registration. Participants are asked to provide background information through an initial inclusion survey, and are then invited to report weekly on their symptoms. In case of symptoms, questions about healthcare-seeking behaviour were asked. GrippeNet.fr representativeness analysis was conducted for the 2011–2012 season,^25,26^ showing that middle-aged, female, and well-educated individuals were overrepresented, but that those at risk of influenza complications were representative of the general population. Around 6000 participants are involved each year in the study. They complete nearly 4000 weekly surveys per week.

The present study focused on three seasons of the GrippeNet.fr study, between 2012 and 2015. The first season took place from 15 November 2012 (before the onset of the seasonal influenza epidemic) to 21 April 2013 (after the end of the seasonal influenza epidemic); the second season took place from 13 November 2013 to 13 April 2014; and the third season from 13 November 2014 to 14 April 2015. All episodes of ILI identified in weekly surveys were included. The occurrence of ILI was defined by a combination of reported symptoms (a modified version of the European Center for Disease Control’s ILI definition^27^): sudden onset of symptoms; in addition to fever, chills, fatigue, headache, or myalgia; and cough, sore throat, or dyspnoea). Two others ILI definitions were tested for sensitivity analysis and for comparison with the literature: the one used in Paolotti *et al*,^24^ which is close to the WHO guidelines (sudden onset of fever with a measured body temperature of ≥38°C, accompanied by headache or muscle pain, and by cough or a sore throat); and the French Sentinelles network's definition (sudden onset of symptoms with a measured body temperature of ≥39°C and myalgia, accompanied by cough, sore throat, dyspnoea, sneezing, sputum, or rhinitis).^7^

Two consecutive surveys reporting ILI for a given participant were considered to correspond to the same ILI episode, if the surveys were completed within a 15-day period, or unless the individual reported otherwise. Healthcare-seeking behaviour was analysed, including GP visit, specialist visit, emergency visit, hospitalisation, pharmacist advice, internet searching, and drug intake. During the three seasons analysed, multiple ILI episodes per participant were observed. To account for this, determinants associated with GP consultation were estimated using a conditional logistic regression, based on a Generalised Estimated Equations (GEE). Variables with a univariate *P*<0.20 were included in the multivariate analysis.

The analysis was first conducted using data collected during the whole study period (15 November 2012 to 14 April 2015). In addition, a more detailed analysis was performed on data from the last study season (from 13 November 2014 to 14 April 2015) to account for additional variables included specifically for the study, and focusing on healthcare access (having a referring GP, the level of health insurance, time to wait before an appointment with a GP, if the GP had excessive fee, and living in an area with an insufficient coverage of healthcare workers), and health status perception during an ILI episode.

Multivariate analysis by progressive elimination (stepwise backward) and progressive introduction (stepwise forward) of the variables were performed. All statistical analyses were performed using the R software (version 3.1.3).

## Results

From the 231 864 weekly questionnaires transmitted by the GrippeNet.fr cohort during the study period, 6023 ILI episodes were identified.

### ILI and individual characteristics

Sociodemographic characteristics and health status for individuals with ≥1 ILI episode are listed in Tables 1 and 2. Over 70% were between 18 and 65 years old. There was a sex imbalance, with 66% of the cohort being female, and an educational imbalance, with most of the individuals having a higher education degree (Table 1). Regarding the healthcare access characteristics of ILI cases during the last season, 26 (1.5%) lived in an area with an insufficient coverage of healthcare workers, 1611 (93.3%) notified a referring doctor, and 1692 (98.0%) were covered by the French national health insurance. Concerning their health characteristics, one-third were considered at risk of influenza complications (between 29.2 and 34.4% depending on the season) (Table 2). Coryza, cough, and sore throat were the more frequent ILI symptoms in total across the three seasons (73.5%, 73.2%, and 69.4% respectively) (Table 3). Half of ILI episodes (3005, 50.3%) led to a modification of activities.

**Table 1.** Sociodemographic characteristics of individuals included in the study (an individual may have been involved in 1, 2, or 3 seasons).

	2012–2013, *n* (%)	2013–2014, *n* (%)	2014–2015, *n* (%)
*N*	1756	1424	1726
**Age, years (MD = 9)**			
<18	192 (11.0)	135 (9.5)	173 (10.0)
18–64	1306 (74.6)	1041 (73.2)	1231 (71.4)
≥65	252 (14.4)	247 (17.3)	320 (18.6)
**Female (MD = 0)**	1154 (65.7)	945 (66.4)	1135 (65.8)
**Children in the household (MD = 12)**			
None	973 (55.6)	851 (59.9)	1024 (59.5)
≥1	778 (44.4)	570 (40.1)	698 (40.5)
**Main activity (MD = 0)**			
Full-time or part-time employment	881 (50.2)	722 (50.7)	854 (49.5)
Self-employment	87 (5.0)	66 (4.6)	91 (5.3)
Attending daycare/school/university	246 (14.0)	182 (12.8)	210 (12.2)
Home-maker/retired	433 (24.7)	355 (24.9)	445 (25.8)
Unemployed	55 (3.1)	45 (3.2)	67 (3.9)
Maternity leave	–	–	6 (0.3)
Long-term sick-leave or parental leave	32 (1.8)	25 (1.8)	24 (1.4)
Other	22 (1.2)	29 (2.0)	29 (1.6)
**Socioprofessional characteristics (MD = 249)**			
Professional or office work	698 (41.9)	585 (43.1)	690 (42.3)
Retail, sales, catering, and hospitality	46 (2.8)	30 (2.2)	37 (2.3)
Skilled manual work	31 (1.9)	30 (2.2)	40 (2.4)
Other manual work	18 (1.1)	12 (0.9)	17 (1.0)
Other	86 (5.2)	64 (4.7)	68 (4.2)
Not applicable	788 (47.2)	636 (46.9)	781 (47.8)
**Level of education (MD = 379)**			
Above high school diploma	833 (58.3)	843 (60.3)	1037 (61.0)
High school diploma or equivalent	222 (15.5)	236 (16.9)	275 (16.2)
Below high school diploma	201 (14.1)	194 (13.9)	236 (13.9)
Not applicable (age <16 years)	174 (12.1)	125 (8.9)	151 (8.9)
**Place of residence (MD = 0)**			
Urban	1412 (80.4)	1157 (81.2)	1385 (80.2)
Rural	344 (19.6)	267 (18.8)	341 (19.8)

MD = missing data.

**Table 2.** Health characteristics of individuals included in the study (an individual may have been involved in 1, 2, or 3 seasons).

	2012–2013, *n* (%)	2013–2014, *n* (%)	2014–2015, *n* (%)
*N*	1756	1424	1726
**Colds/flu-like diseases frequency (MD = 69)**			
Rare	1291 (74.5)	1011 (72.1)	1285 (75.5)
Often	421 (24.3)	363 (25.9)	393 (23.1)
Don’t know	20 (1.2)	28 (2.0)	25 (1.4)
**Flu vaccination for the current season (MD = 0)**			
No	1360 (77.5)	1062 (74.5)	1278 (74.0)
Yes	394 (22.4)	361 (25.4)	445 (25.8)
Don’t know	2 (0.1)	1 (0.1)	3 (0.2)
**Smoking status (MD = 0)**			
Never smoked	1019 (58.0)	819 (57.5)	997 (57.8)
Used to smoke	480 (27.4)	408 (28.7)	517 (30.0)
Still smoking	257 (14.6)	197 (13.8)	212 (12.2)
**Comorbidities** (MD = 0)			
No comorbidities	1430 (81.4)	1135 (79.7)	1363 (79.0)
≥1 comorbidity	326 (18.6)	289 (20.3)	363 (21.0)
Treated asthma	131 (7.5)	118 (8.3)	144 (8.3)
Treated diabetes	48 (2.7)	47 (3.3)	48 (2.8)
Treated heart condition	119 (6.8)	90 (6.3)	142 (8.2)
Treated kidney condition	7 (0.4)	10 (0.7)	9 (0.5)
Treated immunodepression	47 (2.7)	40 (2.8)	49 (2.8)
Treated pulmonary condition	50 (2.8)	51 (3.6)	58 (3.4)
≥1 respiratory allergy (MD = 0)	677 (38.6)	566 (39.7)	659 (38.2)
**Pregnancy (MD = 35)**			
Pregnant	7 (0.4)	8 (0.6)	24 (1.4)
Not pregnant	544 (31.3)	424 (29.9)	512 (29.8)
Not applicable (men or women <15 or >50 years old)	1188 (68.3)	984 (69.5)	1180 (68.8)
**BMI, kg/m^2^** (MD = 73)			
<18.5	200 (11.6)	156 (11.1)	190 (11.2)
18.5–29	1348 (78.1)	1081 (76.8)	1296 (76.2)
30–39	158 (9.2)	152 (10.8)	197 (11.5)
≥40	19 (1.1)	19 (1.3)	18 (1.1)
**At risk of influenza complications (MD = 85)**			
No	1215 (70.8)	954 (67.8)	1114 (65.6)
Yes	501 (29.2)	453 (32.2)	584 (34.4)

BMI = body mass index. MD = missing data.

**Table 3.** Characteristics of ILI episodes reported by participants included in the study.

	2012–2013, *n* (%)	2013–2014, *n* (%)	2014–2015, *n* (%)	Total, *n* (%)
*N*	2184	1772	2067	6023
**Symptoms (MD = 0)**				
Fever	1162 (53.2)	820 (46.3)	1057 (51.1)	3039 (50.5)
Chills	950 (43.5)	696 (39.3)	898 (43.4)	2544 (42.2)
Coryza	1578 (72.3)	1309 (73.9)	1538 (74.4)	4425 (73.5)
Sneezing	1206 (55.2)	970 (54.7)	1120 (54.2)	3296 (54.7)
Sore throat	1516 (69.4)	1287 (72.6)	1377 (66.6)	4180 (69.4)
Cough	1606 (73.5)	1256 (70.9)	1549 (74.9)	4411 (73.2)
Dyspnoea	440 (20.1)	355 (20.0)	476 (23.0)	1271 (21.1)
Headaches	1427 (65.3)	1151 (65.0)	1362 (65.9)	3940 (65.4)
Pain	898 (41.1)	637 (35.9)	771 (37.3)	2306 (38.2)
Sputum	436 (20.0)	346 (19.5)	366 (17.7)	1148 (19.1)
Asthenia	1086 (49.7)	809 (45.7)	1008 (48.8)	2903 (48.2)
**Sudden onset of symptoms (MD = 84)**				
Yes	2018 (93.7)	1649 (94.3)	1915 (94.1)	5582 (94.0)
No	136 (6.3)	100 (5.7)	121 (5.9)	357 (6.0)
**Mean health score (± SD) (MD = 94)**	–	–	42.8 (± 21.4)	–
**Activities modification (MD = 50)**				
No	1056 (48.7)	937 (53.4)	975 (47.6)	2968 (49.7)
Yes, without taking time off work/school	662 (30.5)	513 (29.2)	631 (30.8)	1806 (30.2)
Yes, with taking time off work/school	450 (20.8)	306 (17.4)	443 (21.6)	1199 (20.1)

ILI = influenza-like illness. MD = missing data. SD = standard deviation.

### Healthcare-seeking behaviour

Among the 6023 ILI episodes identified over the whole study period, 1961 (32.6%) led to a GP consultation (Table 4). This rate was stable during the 3 years of the study, but varied across French regions, ranging from 27.4% (*n *= 270) to 43.5% (*n* = 62). No significantly different result in GP consultation rate was found for individuals at risk of influenza complications (33.6%, *P* = 0.42). Advice from a pharmacist was asked for in 361 ILI cases (9.4%). An emergency hospital consultation occurred in 142 ILI episodes (2.4%), and a hospitalisation occurred in 57 ILI cases (1.0%). A specialist was consulted in 98 ILI episodes (1.6%). The rate of treatment with influenza antivirals was 0.8% among ILI cases (*n* = 47). This rate was not significantly different between ILI episodes in individuals at risk of influenza complications (0.8%) and those in individuals without risk factors (0.7%).

**Table 4.** Healthcare-seeking behaviour following ILI in participants included in the study

	2012–2013, *n* (%)	2013–2014,* n* (%)	2014–2015, *n* (%)	Total, *n* (%)
**ILI episodes** *N*	2184	1772	2067	6023
**Consultation (MD = 0)**				
GP	738 (33.8)	535 (30.2)	688 (33.3)	1961 (32.6)
GP consultation among individuals at risk of ILI complications	218 (34.9)	175 (31.0)	244 (34.5)	637 (33.6)
Pharmacist	–	188 (10.6)	173 (8.4)	361 (9.4)
Hospital emergencies	59 (2.7)	39 (2.2)	44 (2.1)	142 (2.4)
Specialist	44 (2.0)	25 (1.4)	29 (1.4)	98 (1.6)
Other	33 (1.5)	19 (1.1)	9 (0.4)	61 (1.0)
None	1425 (65.2)	1137 (64.2)	1303 (63.0)	3865 (64.2)
**Hospitalisations (MD = 60)**	20 (0.9)	16 (0.9)	21 (1.0)	57 (1.0)
**Drug intake (MD = 5)**				
Analgesics	1904 (87.2)	1508 (85.3)	1806 (87.4)	5218 (86.7)
Antitussives	815 (37.3)	565 (32)	767 (37.1)	2147 (35.7)
Antibiotics (prescribed)	451 (20.7)	328 (18.6)	417 (20.2)	1196 (19.9)
Antibiotics (self-medication)	31 (1.4)	25 (1.4)	25 (1.2)	81 (1.3)
Antivirals (prescribed)	18 (0.8)	11 (0.6)	18 (0.9)	47 (0.8)
Antivirals (self-medication)	1 (0.0)	2 (0.1)	2 (0.1)	5 (0.1)
Antivirals prescribed to individuals at ILI risk	4 (0.6)	5 (0.9)	7 (1.0)	16 (0.8)
Homeopathy	400 (18.3)	273 (15.4)	340 (16.5)	1013 (16.8)
Aromatherapy or herbal medicine	–	373 (21.1)	414 (20.0)	787 (20.5)
Others	499 (22.8)	366 (20.7)	435 (21.1)	1300 (21.6)
**Internet search (MD = 73)**	145 (6.7)	125 (7.1)	187 (9.1)	457 (7.7)

ILI = influenza-like illness. MD = missing data

Using Paolotti *et al*'s ILI definition, 716 cases (52.8%) led to a GP consultation, and using Sentinelles network ILI definition, 259 cases (56.7%) led to a GP consultation.

### Associated determinants

Focusing on sociodemographic variables (Table 5), a positive association was found between GP consultation for ILI and living in a rural area (OR = 1.21, 95% CI = 1.02 to 1.43) or having a level of education below high school diploma (OR = 1.43, 95% CI = 1.18 to; 1.74). A positive association was found between GP consultation and symptoms of fever (OR = 2.57, 95% CI = 2.26 to 2.93), cough (OR = 1.60, 95% CI = 1.37 to 1.86), dyspnoea (OR = 1.38, 95% CI = 1.19 to 1.60), sputum (OR = 1.75, 95% CI = 1.50 to 2.05), and asthenia (OR = 1.93, 95% CI = 1.70 to 2.19), and a negative association when sneezing (OR = 0.78, 95% CI = 0.69 to 0.88). Regarding behavioural characteristics, a positive association was found between GP consultation and a previous GP consultation for another ILI episode in the same influenza season (OR = 1.42, 95% CI = 1.11 to 1.83); this was also the case with internet searching in case of ILI (OR = 1.63, 95% CI = 1.30 to 2.03). A negative association was found with the use of alternative medicine (homeopathy, herbal medicine, or essential oils) (OR = 0.68, 95% CI = 0.58 to 0.78).

**Table 5.** Significant factors associated with GP consultation for ILI in France between 2012 and 2015 in multivariate analysis

	*N*	Number of ILI episodes with a GP consultation, *n* (%)	OR (95% CI)	*P-*value
**Level of education**				
Above high school diploma	3312	972 (29.3)	1 (Ref)	
High school diploma or equivalent	895	287 (32.1)	1.25 (1.03 to 1.52)	0.021
Below high school diploma	780	293 (37.6)	1.43 (1.18 to 1.74)	<0.001
Not applicable (age <16 years)	579	266 (45.9)	1.63 (1.30 to 2.03)	<0.001
**Residence**				
Urban	4859	1554 (32.0)	1 (Ref)	
Rural	1164	407 (35.0)	1.21 (1.02 to 1.43)	0.026
**Internet search in case of ILI**				
No	5493	1733 (31.5)	1 (Ref)	
Yes	457	210 (46.0)	1.63 (1.30 to 2.03)	<0.001
**Previous GP consultation for ILI during the current influenza season**				
No	5648	1733 (31.4)	1 (Ref)	
Yes	375	188 (50.1)	1.43 (1.11 to 1.83)	0.005
**Alternative medicines utilisation**				
No	4450	1529 (34.4)	1 (Ref)	
Yes	1568	431 (27.5)	0.68 (0.58 to 0.78)	<0.001
S**ymptoms^a^**				
Fever	3039	1350 (44.4)	2.57 (2.26 to 2.93)	<0.001
Sneezing	3296	1042 (31.6)	0.78 (0.69 to 0.88)	<0.001
Cough	4411	1633 (37.0)	1.60 (1.37 to 1.86)	<0.001
Dyspnea	1271	558 (43.9)	1.38 (1.19 to 1.60)	<0.001
Sputum	1148	551 (48.0)	1.75 (1.50 to 2.05)	<0.001
Asthenia	2903	1209 (41.6)	1.93 (1.70 to 2.19)	<0.001

^a^The reference group had no symptoms. ILI = influenza-like illness. OR = odds ratio. Ref = reference.

In the analysis of the 2014–2015 season, when healthcare access characteristics were added, a positive association was found with between GP consultation and having a personal doctor whose costs can be recovered through the national health insurance programme (OR = 2.86, 95% CI = 1.72 to 4.76). There was no statistically significant association between GP consultation and the perception of having to wait a long time to obtain an appointment with a GP, nor with having a complementary healthcare insurance covering most of the consultation fees. A negative perception of own health status during an ILI episode was positively associated with GP consultation (OR = 1.51, 95% CI = 1.08; 2.13). The other results were similar to those observed for the whole study period from 2012 to 2015. The only differences were that the following associations demonstrated in the whole study period were not demonstrated in the 2014–2015 season: between GP consultation and a) living in a rural area, b) having a previous GP consultation for another ILI in the current influenza season, or c) symptoms of sneezing.

## Discussion

### Summary

This study characterised for the first time the healthcare-seeking behaviour for ILI among the general population (and specific subgroups) in France, and identified the factors associated with GP consultation following an ILI episode.

### Strengths and limitations

The prospective nature of the study in the general population — with no other inclusion criteria than living in France, having signed up to the study, and having experienced ≥1 ILI episode — provides a high standard of proof to the results. The use of the GrippeNet.fr cohort allowed the inclusion of many ILI cases, making it possible to use a multivariate analysis. GEEs allowed the link between various ILI episodes experienced by the same individual to be taken into account. However, the GrippeNet.fr population is not fully representative of the French general population. Women, middle-aged individuals, and those with a higher education level and a higher social status are overrepresented. This could mean the real rate of GP consultation for ILI is underestimated, because of the lower consultation rate of highly educated individuals.

### Comparison with existing literature

The rate of GP consultation for ILI in the French general population was 32.6% (*n* = 1961) in this study, lower than in the few previous French studies (57% and 62%).^17,18 ^The first study^17^ was based on a cohort of household contacts, where one member of the household (the index case) had already visited a physician for ILI. It was expected, therefore, that there would be a higher GP consultation rate among secondary ILI cases in these households than in the general population. The second study^18^ was conducted during the A(H1N1)pdm2009 pandemic, therefore higher rates could be explained by the specific epidemic situation. A third study^24^ analysed European data and reported a GP consultation rate over 60% for France. The discrepancy with the results of the current study arises from a difference in the ILI definition criteria used, with the definition used in the third study requiring the measurement of the fever level, where the current study included only incidences of fever as reported by the participants, without a requirement to measure the fever level. Indeed, applying the Paolotti *et al* ILI definition to the current study, the rate of GP consultation for ILI increases to 52.8%. Differences in healthcare systems and access to health care could explain the differences between this study's results and the findings obtained in foreign studies. In France, for example, a patient has to pay €23 for a GP consultation, whereas in the Israeli study all the consultations were free,^12^ and in the US the consultation is ≥US$100. Nevertheless, the GP consultation rate estimated in this study remained in the low average of international studies.^16,24,28^

Trying to reduce this consultation rate could be helpful to keep GPs free to see patients who develop serious illnesses as a result of influenza infection, while also continuing to manage their normal caseload, especially in case of influenza pandemic.^29^ The problem is that individuals at risk of influenza complications did not consult more than the others in this study in case of ILI, in contrast with previous results in the US.^13,14^ This may be due to a difference in public prevention policy, or more likely because of the different health insurance access in the US: some individuals who are >65 years of age, are disabled, or are at risk of influenza complications benefit freely from Medicare. Furthermore, individuals at risk of influenza complications rarely received an antiviral treatment in case of ILI (0.8%), even though it is recommended by the French public health authorities^19^ as well as in most other countries.^20,21^ Pharmacists are not allowed to dispense antiviral medication independently in France; it can be done only with a physician’s prescription. This highlights that individuals at risk of influenza complications, as a group, are not supported correctly and should be encouraged to consult in cases of ILI.

The association found between GP consultation and lower educational level may indicate a higher self-care autonomy in individuals with a higher educational level, leading to different healthcare utilisation.^30^ In contrast, this study found that searching information on the internet during an ILI episode, which could be a sign of needing to increase awareness of self-care, is positively associated with health-seeking behaviour. In terms of clinical presentation, this study found that a large set of symptoms preferentially led to consulting a physician, as already shown in other studies.^17,18^ Additionally, a negative perception of one's own health was found to be positively associated with healthcare-seeking behaviour, as is already well documented.^31,32^

With regard to healthcare access, the only barrier found was not having a personal doctor, which is in accordance with existing literature.^23^ Maintaining close links with a personal doctor seems to facilitate consultations. Similarly, a person who has already consulted in the previous year for an ILI episode is more likely to visit the GP again for a new episode. Not using alternative medicine (homeopathy, herbal medicine, or essential oils) for ILI is positively associated with GP consultation, as it likely indicates trust in and adherence to conventional medicine. Other barriers identified in previous studies — such as financial issues (health insurance^33 ^or excess fees) and geographical considerations, such as density of GPs per inhabitant^34,35^ — were not associated with a reduction of GP consultation in this study. Differences in the healthcare system, access, and costs in other countries may be the reason behind these differing results. This study found that the unemployed population in France did not consult less than the general population, unlike in the US.^13^ Finally, some studies have already shown that women have a higher rate of healthcare use than men,^34^ and this association was also found in this study.

### Implications for research and practice

These results, obtained using data collected directly from the general population, will help practitioners, researchers, and health authorities to have a better understanding of influenza's disease burden during a winter season. It will be very useful in the future to compile the rate of GP consultation in case of ILI estimated in this study with the data collected every year by the French GPs' surveillance networks to estimate ILI incidence in the general population during influenza epidemics. These parameters could also be integrated into influenza mathematical model to improve their accuracy.

Characterising further healthcare-seeking behaviour of the general population in case of ILI and factors associated with a GP consultation would seem to be essential in order to characterise healthcare accessibility. It will also be helpful to improve influenza prevention campaigns. Evaluation of healthcare-seeking behaviour in specific groups of individuals, such as those at risk of influenza complications, is a way to ensure that they have the opportunity to receive appropriate treatment. An influenza antiviral treatment is recommended by the French public health authorities in this population in case of ILI.^19^
